# The Global Theranostics Access Gap: Real-World Disparities in PSMA PET/CT and ^177^Lu-PSMA Therapy Between Centers in Brazil and Europe

**DOI:** 10.2967/jnumed.126.273029

**Published:** 2026-11

**Authors:** Juliano J. Cerci, Stefano Fanti, Ken Herrmann, Denise Ferreira Rodrigues, Pedro Henrique Furst Leite, Margaret Masukawa, Marcelo Moreira da Silva, Wolfgang P. Fendler, Adriano Goncalves e Silva, Elise Nara Sanfelice, Walter Ayelet, Fabio Ghetti, Andrea Bascioni, Enrique E. Estrado Lobato, Diana Paez

**Affiliations:** 1 Instituto Teranóstico Brasil, Curitiba, Brazil;; 2 IRCCS Azienda Ospedaliero-Universitaria di Bologna, Bologna, Italy;; 3 Department of Nuclear Medicine, West German Cancer Center, DKTK and NCT Site, University Hospital Essen, Essen, Germany;; 4 Department of Nuclear Medicine, IMEB–Institute of Nuclear Medicine of Brasília, Brasília, Brazil;; 5 Instituto do Cancer e Transplante, Curitiba, Brazil;; 6 CIONC, Curitiba, Brazil; and; 7 Division of Human Health, International Atomic Energy Agency, Vienna, Austria

**Keywords:** access gap, radionuclide therapy, ^177^Lu-PSMA, prostate cancer, radiopharmaceutical therapy, theranostics

## Abstract

Real-world disparities in access to PSMA-targeted theranostics are poorly characterized. We compared waiting times for PSMA PET/CT and ^177^Lu-PSMA therapy between centers in Brazil and Europe. **Methods:** This retrospective multicenter study included 91 patients (50 from Brazil, 41 from Europe) treated with ^177^Lu-PSMA. Outcomes were days from request to PSMA PET/CT completion (diagnostic access) and days from request to first therapy dose (therapeutic access). Groups were compared using the Mann–Whitney *U* test. **Results:** Diagnostic access was similar between Brazil (median, 17.5 d; interquartile range [IQR], 9–34 d) and Europe (median, 19 d; IQR, 14–23 d; *P* = 0.39). Therapeutic access was significantly delayed in Brazil (median, 59 d; IQR, 54.5–91) compared with Europe (median, 21 d; IQR, 17–31 d; *P* < 0.01), representing a 2.8-fold longer wait. **Conclusion:** Despite comparable access to PSMA PET/CT, initiation of ^177^Lu-PSMA therapy is nearly 3 times slower in Brazil than in Europe. Closing the therapeutic access gap requires targeted investments in radiopharmaceutical supply chains and regulatory efficiency.

## Visual Abstract

**Figure:**
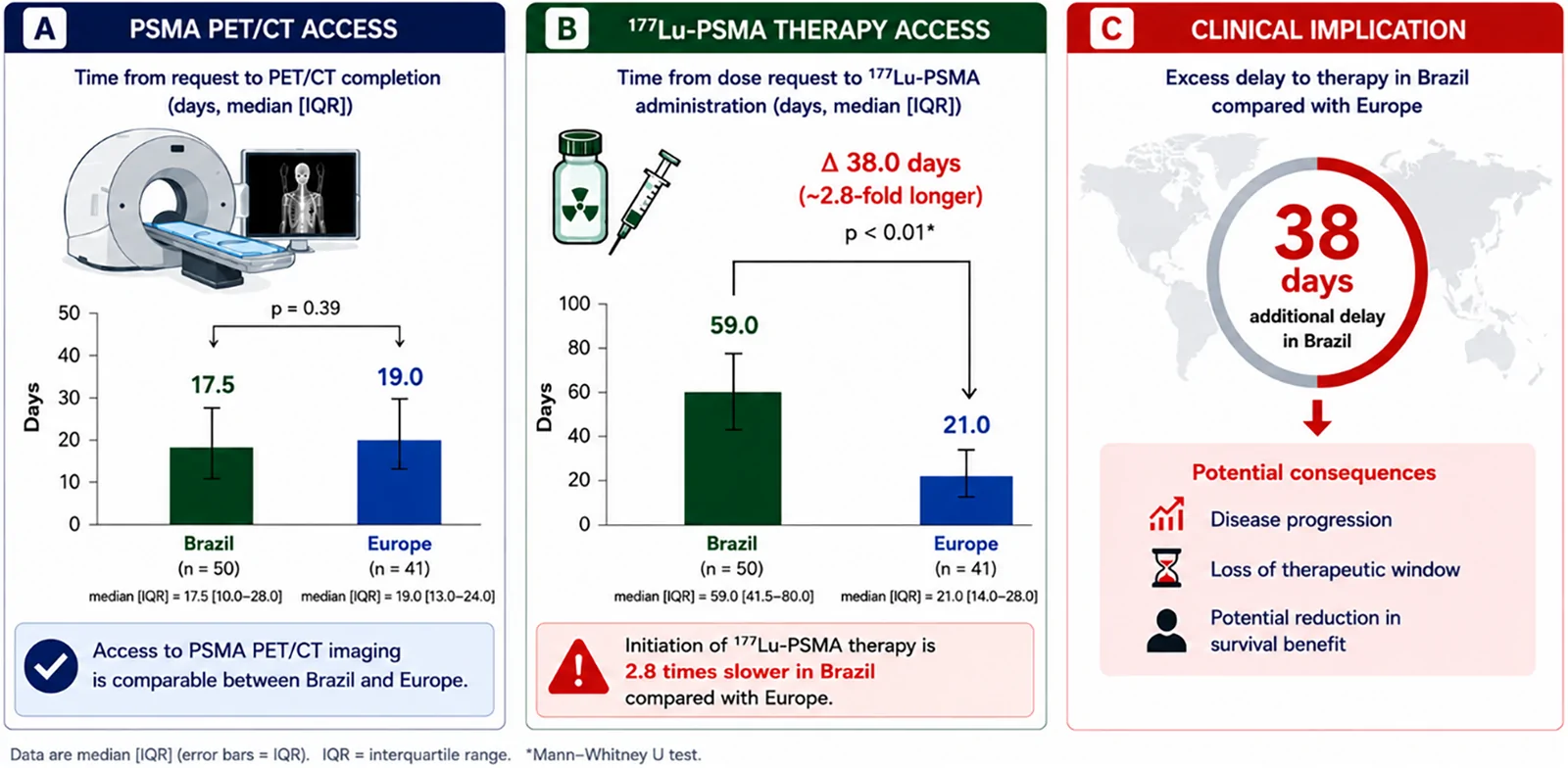

PSMA-targeted radiopharmaceutical therapy with ^177^Lu-vipivotide tetraxetan (Pluvicto; Novartis) improves survival in patients with metastatic castration-resistant prostate cancer (mCRPC) (1,2). PSMA PET/CT has also demonstrated substantial clinical value across initial staging and biochemical recurrence settings (3,4). However, real-world access to theranostics—both diagnostic PSMA PET/CT and subsequent therapy—may vary substantially across regions. Comparative data on access disparities remain scarce, limiting efforts to achieve equitable precision oncology care globally. In this study, we evaluated the regional disparities in access to PSMA PET/CT imaging and the initiation of ^177^Lu-PSMA radiopharmaceutical therapy between centers in Brazil and Europe.

## MATERIALS AND METHODS

This retrospective, multicenter, real-world analysis included consecutive patients treated with PSMA-targeted radiopharmaceutical therapy at 4 centers in Brazil (50 patients) and 2 centers in Europe (Italy and Germany; 41 patients). Two additional Brazilian patients were excluded because they died after therapy was requested but before treatment initiation. Data collected included age, disease duration, number of prior lines of systemic therapy, time from PSMA PET request to PET acquisition, and time from therapy request to first treatment administration. Waiting times were summarized as median and interquartile range (IQR). Comparisons between regions were performed using the Mann–Whitney *U* test. *P* values of less than 0.05 were considered statistically significant.

## RESULTS

Ninety-one patients were included in the study. Baseline characteristics were similar between groups. The median age was 74.0 y (Brazil) versus 73.7 y (Europe), and both groups had received a median of 3 prior therapy lines (Table 1).

**TABLE 1.** Clinical and Access Characteristics

Variable	Brazil	Europe
*n*	50	41
Age (y)	74.0 (66.6–80.2)	73.7 (67.4–79.1)
Disease duration (y)	8.9 (5.0–13.6)	4.7 (2.0–7.4)
No. prior therapy lines	3 (2–3)	3 (3–3)
Time from PSMA PET/CT request to scan completion* (d)	17.5 (9.0–34.0)	19.0 (14.0–23.0)
Time from ¹^77^Lu-PSMA therapy request to dose administration† (d)	59.0 (54.5–91.0)	21.0 (17.0–31.0)

* *P* = 0.39.

† *P* < 0.01.

Data expressed as median, followed by IQR in parentheses.

Access to PSMA PET/CT imaging was comparable between regions. The median waiting time from scan request to scan completion was 17.5 d (IQR, 9.0–34.0 d) in Brazilian centers and 19.0 d (IQR, 14.0–23.0 d) in European centers (*P* = 0.39). Brazilian centers showed wider variability (5–61 d vs. 2–73 d in Europe).

Substantial disparities were observed for therapeutic access. The median interval from dose request to first ^177^Lu-PSMA administration was 59.0 d (IQR, 54.5–91.0 d) in Brazil and 21.0 d (IQR, 17.0–31.0 d) in Europe (*P* < 0.01). This represents a 2.8-fold longer median delay in Brazil, with an absolute median difference of 38 d (Fig. 1).

**FIGURE 1.**
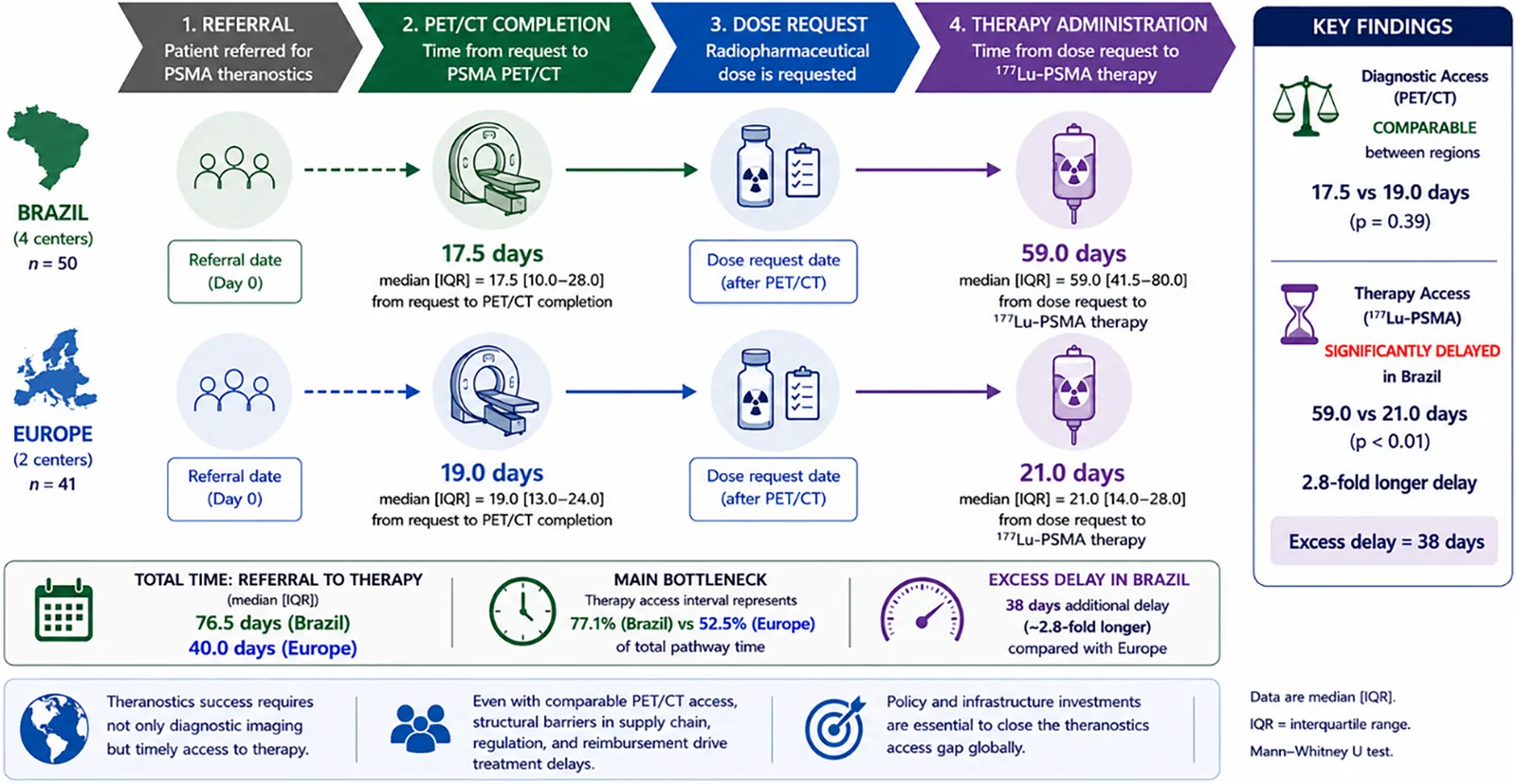
Theranostics access cascade: real-world timelines from referral to ^177^Lu-PSMA therapy in Brazil versus Europe.

## DISCUSSION

To our knowledge, this is the first multicenter real-world analysis quantifying disparities in both diagnostic and therapeutic access to PSMA-targeted theranostics between Brazil and Europe. Our findings reveal a striking asymmetry within the theranostic pathway: while PSMA PET/CT access is comparable (median, 17.5 d vs. 19.0 d), access to radiopharmaceutical therapy is profoundly delayed in Brazil (median, 59.0 d vs. 21.0 d).

The comparable waiting times for PSMA PET/CT between Brazil and Europe may be partly explained by industry-supported access programs. In Brazil, Novartis provides free PSMA PET/CT imaging for eligible patients, bypassing insurance and reimbursement barriers. This model expedites diagnostic access. In contrast, ^177^Lu-PSMA therapy remains subject to complex authorizations and radiopharmaceutical logistics, contributing to the observed treatment disparities. Real-world studies have demonstrated that treatment outcomes and access may also be influenced by patient characteristics, institutional experience, treatment capacity, and radiopharmaceutical logistics (5–8). In addition, the economic implications of ^177^Lu-PSMA therapy have been evaluated in European health care systems, further emphasizing the importance of structured reimbursement and implementation pathways (9).

The clinical implications of our findings are substantial. In the VISION trial, the median overall survival in patients treated with ^177^Lu-PSMA-617 was 15.3 mo (∼465 d) (1). The additional 38-d delay observed in Brazil corresponds to approximately 8% of this survival window. The exclusion of 2 Brazilian patients who died awaiting therapy further underscores the urgency of addressing these delays.

Structural factors likely contributing to this disparity include regulatory approval processes for individual treatment cycles, insurance authorization requirements, and limited capacity of the radiopharmaceutical supply chain. European centers benefit from mature regulatory frameworks, structured reimbursement pathways, and proximity to production facilities.

Limitations of this study include the small number of centers (*n* = 6), retrospective design, potential selection bias from excluded patients, and baseline differences in disease duration (median, 8.9 y in Brazil vs. 4.7 y in Europe), which may reflect referral patterns but does not explain the magnitude of the therapeutic access gap. Importantly, the consistency and magnitude of the disparity (2.8-fold longer delay) suggest a robust inequity.

## CONCLUSION

Although access to diagnostic PSMA PET/CT imaging is comparable between Brazil and Europe, initiation of ^177^Lu-PSMA therapy is nearly 3 times slower in Brazil. This treatment access gap represents a significant global inequity in precision oncology. Structural investments in radiopharmaceutical production capacity, regulatory streamlining, and treatment infrastructure are urgently needed to ensure equitable access to PSMA-targeted radiopharmaceutical therapy worldwide.

## DISCLOSURE

Juliano Cerci reports fees from AstraZeneca, Bayer, GE HealthCare, Janssen, Novartis, Merck, and Takeda Pharma. Wolfgang Fendler reports fees from SOFIE Biosciences, Janssen, Perceptive, Bayer, Novartis, Telix, GE HealthCare, Eczacıbaşı Monrol, Abx, Amgen, Urotrials, Lilly, AstraZeneca, Curium, and Bristol Myers Squibb. Ken Herrmann reports fees from Bayer, Janssen, Novartis, and Takeda Pharma. No other potential conflict of interest relevant to this article was reported. The AI tool DeepSeek was used to polish writing but not to generate data.

## Notes

KEY POINTS **QUESTION:** Are there differences in real-world access to PSMA PET/CT imaging and ^177^Lu-PSMA radiopharmaceutical therapy between centers in Brazil and Europe? **PERTINENT FINDINGS:** In this multicenter retrospective cohort study of 91 patients from 4 Brazilian and 2 European centers, access to PSMA PET/CT imaging was found to be similar between regions. However, the median time to ^177^Lu-PSMA therapy was 59 days in Brazil compared with 21 days in Europe, representing a statistically significant 2.8-fold difference. **IMPLICATIONS FOR PATIENT CARE:** Substantial delays in radiopharmaceutical therapy delivery may limit the benefits of precision oncology and highlight the need for improved access pathways for patients eligible for PSMA-targeted treatment.
